# New evidence for the east–west spread of the highly pathogenic avian influenza H5N1 virus between Central Asian and east Asian-Australasian flyways in China

**DOI:** 10.1080/22221751.2019.1623719

**Published:** 2019-06-05

**Authors:** Weiyue Meng, Qiqi Yang, Bram Vrancken, Zhuo Chen, Dongping Liu, Lixia Chen, Xiang Zhao, Sarah François, Tian Ma, Ruyi Gao, Wendong Ru, Yunfeng Li, Hongxuan He, Guogang Zhang, Huaiyu Tian, Jun Lu

**Affiliations:** a Research Institute of Forest Ecology, Environment and Protection, Chinese Academy of Forestry, Key Laboratory of Forest Protection of State Forestry and Grassland Administration, National Bird Banding Center of China, Beijing, People’s Republic of China; b State Key Laboratory of Remote Sensing Science, College of Global Change and Earth System Science, Beijing Normal University, Beijing, People’s Republic of China; c Department of Microbiology and Immunology, Rega Institute, Laboratory of Evolutionary and Computational Virology, KU Leuven, Leuven, Belgium; d National Institute for Viral Disease Control and Prevention, Collaboration Innovation Center for Diagnosis and Treatment of Infectious Diseases, Chinese Center for Disease Control and Prevention, Key Laboratory for Medical Virology, National Health and Family Planning Commission, Beijing, People’s Republic of China; e Department of Zoology, University of Oxford, Oxford, UK; f Swan Lake Wetland Park Management Division of Sanmenxia City, Henan, People’s Republic of China; g National Research Center for Wildlife Borne Diseases, Institute of Zoology, Chinese Academy of Sciences, Beijing, People’s Republic of China

**Keywords:** H5N1 virus, Bird migration, Central Asian flyway, east Asian-Australasian flyway, Spatial temporal analysis

## Abstract

The spread of highly pathogenic avian influenza (HPAI) H5N1 virus is associated with wild fowl migration in East Asian-Australasian (EA) and Central Asian (CA) flyways. However, the spread of H5N1 virus between the two flyways is still unclear. Here, the movements of wild waterfowl were obtained from satellite tracking data covering seven bar-headed geese and three great black-headed gulls breeding in the Qinghai Lake area (along the EA flyway), and 20 whooper swans wintering in the Sanmenxia Reservoir area (at the CA flyway). From the 2688 samples that were screened from wild birds at Qinghai Lake after an outbreak of H5N1 in July 2015, four genomes of H5N1 virus were obtained from bar-headed geese. The results of phylogenetic analysis indicated that these H5N1 viruses belonged to clade 2.3.2.1c and their gene fragments were highly homologous with A/whooper swan/Henan/SMX1/2015 (H5N1) virus (ranging from 99.76% to 100.00%) isolated from a dead whooper swan from the Sanmenxia Reservoir area along the EA flyway in January 2015. Furthermore, the coincidental timing of the H5N1 outbreak with spring migration, together with phylogenetic evidence, provided new evidence of the east-to-west spread of HPAI H5N1 between the EA and CA migratory flyways of China.

## GRAPHICAL ABSTRACT

**Figure:**
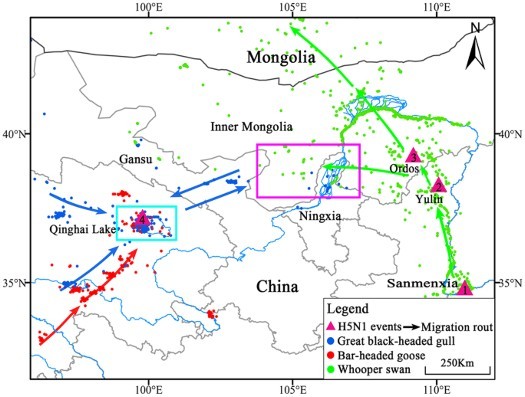

In 2005, there was an outbreak of highly pathogenic avian influenza (HPAI) H5N1 in wild bird populations at Qinghai Lake in west China along the Central Asian (CA) flyway. More than 6,000 wild birds breeding at the lake died, causing considerable public concern [1,2]. Ten years later, in January 2015, 96 waterfowl, including whooper swans (*Cygnus cygnus*) and common pochard (*Aythya ferina*) wintering in Sanmenxia in mid-China along the East Asian-Australasian (EA) flyway died from H5N1 [3]. The timing of these H5N1 outbreaks and the presence of the virus are closely associated with migration of wildfowl within flyways [4]. A previous study detected that H5N1 isolated in central China was genetically similar to H5N1 isolated from Poyang Lake in southeast China [5], suggesting H5N1 transmission between migratory birds in eastern and western China. Here, we provide new evidence on the potential existence of an east-to-west pathway of H5N1 spread between the EA and CA flyways in China.

The migration routes of seven bar-headed geese (*Anser indicus*) and three great black-headed gulls (*Larus ichthyaetus*) breeding in the Qinghai Lake area and 20 whooper swans wintering in Sanmenxia were tracked by satellite from 2006 to 2015 [6–8] (Table S1). After an outbreak of H5N1 in July 2015, a total of 2688 samples were collected from wild birds around Qinghai Lake. Patterns in both datasets were analysed to identify potential between-flyway movements of HPAI H5N1 in China (Supplementary Materials).

Four viruses were successfully recovered from 2688 samples (Table S2): A/bar-headed goose/Qinghai/F/2015, A/bar-headed goose/Qinghai/47/2015, A/bar-headed goose/Qinghai/70/2015, and A/bar-headed goose/Qinghai/133/2015. The hemagglutinin (HA) protein cleavage sites from all four of these isolates had the QRERRRKR amino acid sequence that is consistent with HPAI virus [9]. All sequences contained four potential glycosylation sites: 31 (MNS), 96 (NLT), 260 (NGT), and 369 (NLT). These sites may affect the viral pathogenicity [10]. No Q226L or G228S substitution was observed, indicating that the isolates had typical poultry sialic acid α-2,3-galactose receptor binding tropism. The neuraminidase (NA) gene contained a 60-nucleotide deletion from position 168–227, resulting in a 20-amino acid deletion (position 49–68) in the stem of the NA protein, which may increase avian influenza virus (AIV) virulence [5]. Mutations occurred at positions N30D and T215A of the matrix protein, which has been shown to increase influenza viral replication ability in mice [11]. The nonstructural protein had a 5-amino acid residue deletion (position 80–84), and the amino acid at position-42 had mutated to serine (Table S3), which correlates with interferon resistance in influenza virus.

Phylogenetic analysis showed that the HA genes from the four isolates belonged to clade 2.3.2.1c with high homology (99.8%), with the highest homology being to A/whooper swan/Henan/SMX9/2015 virus. The HA gene sequences of the four isolates fell within the same cluster with A/whooper swan/Shanxi/17L/2015 (Figure 1(a)). The NA genes of the four isolates were located in the same clade of the phylogeny, with 99.9–100% nucleotide homology, and shared the highest homology (99.9–100%) with A/whooper swan/Henan/SMX9/2015 isolated in Sanmenxia in eastern China. The NA gene sequences of the four viruses were closely related to A/chicken/Jiangsu/2477/2014 and A/pigeon/Zhejiang/112090/2014, isolated from east China (Figure S1). The internal genes from the four isolates were highly homologous (99.76–100%) and clustered with A/whooper swan/Henan/SMX1/2015 (H5N1) (high homology ranging from 99.76–100%) (Figures S2–S7). The internal genes from the four isolates were closely related to those isolated from whooper swans from Sanmenxia and black-necked grebes from Inner Mongolia along the EA flyway in 2015. They were also closely related to the isolates from east China (Jiangsu and Zhejiang) in 2014 (Table S4).

**Figure 1.**
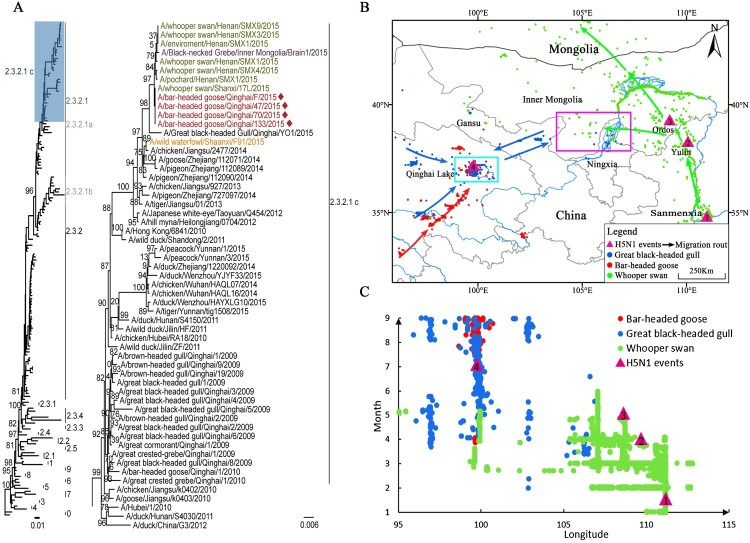
Phylogenetic analysis of the HA gene migratory routes and sampling locations for wildfowl. a. Maximum-likelihood phylogenetic tree of HPAI H5N1 viral HA sequences and the subtree of clade 2.3.2.1c. Clade 2.3.2.1c of the H5N1 viruses is highlighted blue. New isolates at Qinghai Lake from this study are shown in red and indicated by the symbol, ♦. Viruses previously isolated from the Sanmenxia Reservoir area are shown in green. The virus isolated from Inner Mongolia is shown in purple. The virus isolated from Yulin, Shaanxi, is shown in orange. b. Migratory routes of three wildfowl species. Numbers 1, 2, 3, and 4 represent the sites where the H5N1 viruses were sampled. The purple box indicates the intersection area of the great black-headed gulls and the whooper swans from March to May (i.e. central Gansu, western Inner Mongolia, and northern Ningxia). The blue box indicates the intersection area of the great black-headed gulls and the bar-headed geese (i.e. western and northern Qinghai Lake from May to August, including Dandao, Buha River Estuary, Tiebuqia River Estuary, Quanwan, and Heima River Estuary). c. Spring migration of three wildfowl species and H5N1 outbreaks from east to west. Circles represent the time and longitude coordinates of the migratory birds: great black-headed gulls (blue), bar-headed geese (red), and whooper swans (green). Purple triangles represent the time and longitude coordinates of H5N1 outbreaks in Sanmenxia, Yulin, Ordos, and Qinghai Lake.

In January 2015, H5N1 virus was detected from whooper swans that were wintering in Sanmenxia (Henan province, site 1) [8]. During the spring migration from mid-February to mid-March, the whooper swans migrated to Yulin (Shaanxi province, site 2), and H5N1 virus was detected in Yulin in April. From mid-February to late April, whooper swans migrated to Ordos (Inner Mongolia province, site 3), and H5N1 virus was detected in Ordos in May (Figure 1(b and c)). The HA genes isolated from whooper swans from Sanmenxia shared similar gene sequences to the Shaanxi and Inner Mongolia isolates (Figure 1(a)). The detection of H5N1 virus was in line with the whooper swans’ migratory routes and timings from Sanmenxia to Ordos. Whooper swans also migrated to northern Ningxia, western Inner Mongolia, and western Gansu in April during the spring migration. Great black-headed gulls from Qinghai also remained in that area from March to May after which they migrated back to Qinghai Lake (site 4) as early as April. From April to September, bar-headed geese often mixed with great black-headed gulls while resting and foraging at important estuaries in Qinghai Lake, and the two species had an overlapping home range and time at Qinghai Lake (Figure 1(b and c)). Strains isolated from four bar-headed geese in Qinghai Lake shared a sequence identity of 99.69–100% with those isolated from whooper swans in Sanmenxia. A discrete phylogeographic analysis was used to corroborate whether the timing of the east-to-west migration is compatible with the proposed sequence of transmission events based on the telemetry data (Figure S8). Reassuringly, the results showed that the posterior density of migration events towards the Qinghai Lake region in the clade of interest took place between November 2014 and June 2015.

The EA and CA flyways are two important migratory routes covering most of China. Sanmenxia in eastern China on the EA flyway is an important wintering ground for whooper swans [12]. The whooper swan is a widely distributed bird in eastern China and the main carrier of AIV. Qinghai Lake along the CA flyway is an important breeding area for migratory birds, such as bar-headed geese and great black-headed gulls, which are dominant species on the CA flyway due to their abundance and wide distribution in the Qinghai–Tibet plateau in western China. In 2005, bar-headed geese and great black-headed gulls were the birds in which the virus was most frequently detected and accounted for most of the wildfowl deaths in the outbreak at Qinghai Lake. The complete genome sequences of four isolates from Qinghai Lake were closely related and highly homologous to those isolated from whooper swans in Sanmenxia and black-necked grebes in Inner Mongolia in 2015. The H5N1 virus may have spread from Sanmenxia to the upper and middle reaches of the Yellow River, along the whooper swans’ migratory route, then being transmitted to great black-headed gulls in the upper and middle reaches of the Yellow River. The virus was then carried to western China along the great black-headed gulls’ migratory route and spread to bar-headed geese breeding at Qinghai Lake in western China.

Our results are highly suggestive of virus transmission from Sanmenxia to Qinghai Lake between two global migratory bird flyways via the wild birds’ migration. Wild bird migration promotes virus transmission between the east and west regions of China (Figure 1). As transportation and trade promoted AIV spread among poultry [13], wild bird migration promotes viral spread between regions. Therefore, during the migratory season, important migratory sites should be closely monitored to reduce the infection risk to poultry and humans.

## Supplementary Material

Supplemental Material
