# Supplementary material for: New evidence for the east–west spread of the highly pathogenic avian influenza H5N1 virus between Central Asian and east Asian-Australasian flyways in China
Source: Emerg Microbes Infect. 2019 Jun 5;8(1):823–6. doi: 10.1080/22221751.2019.1623719 (PMC6567254; doi:10.1080/22221751.2019.1623719)
Supplement: Supplemental Material [file TEMI_A_1623719_SM1171.zip › Supplementary Material/temi-2018-0039-20190522173938/doc/Supplementary material final.docx]

**Supplementary materials**

**AIV isolation, identification, and gene mutation analysis**

Samples were collected from five locations including the Heima River Estuary, Buha River Estuary, Tiebuqia River Estuary, Quanwan, and Niaodao Island in Qinghai Lake, from June 28 to July 10, 2018. Fresh feces samples, tissues, and organs from dead bar-headed geese were collected and refrigerated at ˗80°C.

The specimens were inoculated into the allantoic cavities of specific-pathogen-free chicken embryos aged 9–10 days, and after culturing for 24–72 h, the chicken embryo allantoic fluid was collected and subjected to a hemagglutination assay. Viral RNA was extracted from positive samples and the presence of AIV was confirmed by reverse transcriptase-polymerase chain reaction (RT-PCR) assays.

The method described above was used to obtain AIV cDNA for identification purposes, and specific primers and universal primers were used for subtype identification. Primer 5.0 (http://www.primer-e.com/primer5_updates.htm) was used to design the primer sequences, and eight fragments were amplified simultaneously for AIV. PCR products were separated by 1% agarose gels for electrophoretic examination. PCR products were recovered in accordance with the kit instructions (Beijing TransGen Biotech), and a commercial company (The Beijing Genomics Institute BGI) performed the DNA sequencing.

**Phylogenetic analysis**

Gene sequences of interest obtained from 1996 to 2018 were retrieved from NCBI GenBank and the Global Initiative on Sharing Avian Influenza Data (GISAID) on June 20, 2018. The HA gene sequences of H5N1 virus used in this study are provided in Table S4. Maximum likelihood phylogenetic trees of HA segment (Figure 1A), NA segment (Figure S1), and internal gene segments (Figure S2–S7) were built using FastTree v2.1.9 ^1^.

Phylogenetic trees were estimated using the Bayesian evolutionary analysis by sampling trees (BEAST) software ^2^. The substitution process was modeled according to the HKY+Γ model ^3^ ^4^. As tests with the exponential growth model showed that the growth rate was indistinguishable from zero (the 95% highest posterior density (HPD) interval of the exponential growth rate encompassed zero), the population size was modeled using a constant function. An uncorrelated log-normal relaxed molecular clock model was used to convert the branch lengths into time units. The significance of the temporal signal was verified by comparing the posterior evolutionary rate estimate obtained from correct sampling times against the null distribution obtained by randomly permutating the sampling dates. For the null estimate, we averaged all possible randomizations in a single analysis ^5^. The migration history was inferred using a model that allows for different rates of migration to and from locations ^6^, and the timing of migration events was estimated using stochastic mapping techniques ^7^ ^8^.

**Satellite telemetry information**

In Qinghai Lake, seven bar-headed geese and three great black-headed gulls were captured in July 2006–2007 during the post-breeding period ^9^, and the locations were acquired from the data collection system (ARGOS 2007). Twenty whooper swans were captured in February 2015 in Sanmenxia, and the location data from Global Positioning System (GPS) were acquired from the China Mobile Communication System at one location in 2-h intervals (Table S1). The two types of transmitters, with similar location accuracy of <150 m of satellite data, were used to determine the migration routes.

Based on the satellite data, whooper swans stayed in Sanmenxia from November to February of the next year, then they passed through Shaanxi in mid-February or mid-March, and stopped at the Yulin wetlands of Shaanxi for a longer time. The whooper swans stayed in Ordos of western Inner Mongolia from the middle of February to the end of April. Some whooper swans migrated to eastern Gansu from February 21 to March 19. Great black-headed gulls departed from Bengal Bay, and most migrated to Qinghai Lake as early as April, but some migrated to northern Ningxia and eastern Gansu in March and returned to Qinghai Lake in May. Bar-headed geese departed from Yarlung Zangbo River of Tibet, then stayed in Qinghai Lake from March to September. Bar-headed geese and great black-headed gulls had overlapping home ranges at Heima River Estuary, Buha River Estuary, Tiebuqia River Estuary, Quanwan, and Niaodao Island in Qinghai Lake from April to August. During spring migration, the three species migrated to the interaction area between northern Ningxia, eastern Gansu, and western Inner Mongolia, and resided in the same area from March to April.

**Ethics statements**

Approval for swan capture was granted by the State Forestry Department of Henan Province (No. 13 Yu Forest Protection [2017]). All animal studies were conducted in strict accordance with the guidelines for animal welfare issued by the World Organization for Animal Health. All experiments were performed in a biosafety level 2+ laboratory (an enhanced animal biosafety level 2 laboratory and a negative pressure-ventilation laboratory). Experimental protocols involving animals were approved by our institution’s animal care committee.

**References**

1 Price, M. N. et al. FastTree 2-approximately maximum-likelihood trees for large alignments. *Plos One*, doi:10.1371/journal.pone.0009490 (2010).

2 Suchard, M. A. et al. Bayesian phylogenetic and phylodynamic data integration using BEAST 1.10. *Virus Evolution* **4**, vey016 (2018).

3 Hasegawa, M. et al. Dating of the human-ape splitting by a molecular clock of mitochondrial DNA. *Journal of Molecular Evolution* **22**, 160-174 (1985).

4 Yang, Z. et al. Comparison of models for nucleotide substitution used in maximum-likelihood phylogenetic estimation. *Molecular Biology and Evolution* **11**, 316-324 (1994).

5 Nídia Sequeira, T. O. et al. Bayesian Inference Reveals Host-Specific Contributions to the Epidemic Expansion of Influenza A H5N1. *Molecular Biology and Evolution* **32**, 3264-3275 (2015).

6 Edwards, C. J. et al. Ancient hybridization and an Irish origin for the modern polar bear matriline. *Current Biology* **21**, 1251-1258 (2011).

7 Minin, V. N. et al. Fast, accurate and simulation-free stochastic mapping. *Philosophical Transactions of the Royal Society of London* **363**, 3985-3995 (2008).

8 Minin, V. N. et al. Counting labeled transitions in continuous-time Markov models of evolution. *Journal of Mathematical Biology* **56**, 391-412 (2008).

9 Zhang, G. G. et al. The Current Status of Waterbirds after Avian Influenza Outbreak at Qinghai Lake,China. *Chinese Journal of Zoology* **43**, 51-56 (2008).
