# Supplementary material for: New evidence for the east–west spread of the highly pathogenic avian influenza H5N1 virus between Central Asian and east Asian-Australasian flyways in China
Source: Emerg Microbes Infect. 2019 Jun 5;8(1):823–6. doi: 10.1080/22221751.2019.1623719 (PMC6567254; doi:10.1080/22221751.2019.1623719)
Supplement: Supplemental Material [file TEMI_A_1623719_SM1171.zip › Supplementary Material/temi-2018-0039-20190522173938/doc/Supplementary Tables final.zip!/Supplementary Table S1.docx]

**Table S1 Satellite tracking of wild waterfowl**

| Species | PTT ID | Capture date | Capture sites | Num. of location |
| --- | --- | --- | --- | --- |
| Bar-headed goose | 66196 | 2006.07.10 | Quanwan | 61 |
| Bar-headed goose | 66197 | 2006.07.19 | Garila | 115 |
| Bar-headed goose | 66199 | 2006.07.18 | Buhahekou | 93 |
| Bar-headed goose | 66200 | 2006.07.21 | Quanwan | 220 |
| Bar-headed goose | 39615 | 2007.07.27 | Garila | 210 |
| Bar-headed goose | 39756 | 2007.07.29 | Garila | 135 |
| Bar-headed goose | 39760 | 2007.07.30 | Garila | 85 |
| Great black-headed gull | 66187 | 2006.07.07 | Quanwan | 410 |
| Great black-headed gull | 66189 | 2006.07.24 | Buhahekou | 584 |
| Great black-headed gull | 66190 | 2006.07.11 | Sankuaishi | 256 |
| Whooper swan | 1001 | 2015.12.8 | Sanmenxia | 1090 |
| Whooper swan | 2000 | 2015.12.8 | Sanmenxia | 1444 |
| Whooper swan | 2001 | 2015.12.8 | Sanmenxia | 1232 |
| Whooper swan | 1023 | 2015.12.8 | Sanmenxia | 1631 |
| Whooper swan | 1024 | 2015.12.8 | Sanmenxia | 2366 |
| Whooper swan | 2017 | 2015.12.8 | Sanmenxia | 1779 |
| Whooper swan | 2019 | 2015.12.8 | Sanmenxia | 1788 |
| Whooper swan | 2145 | 2015.12.8 | Sanmenxia | 645 |
| Whooper swan | 1006 | 2015.12.8 | Sanmenxia | 1540 |
| Whooper swan | 2137 | 2015.12.8 | Sanmenxia | 1232 |
| Whooper swan | 1048 | 2015.12.8 | Sanmenxia | 1780 |
| Whooper swan | 1020 | 2015.12.8 | Sanmenxia | 1244 |
| Whooper swan | 2018 | 2015.12.8 | Sanmenxia | 5231 |
| Whooper swan | 132100 | 2015.12.8 | Sanmenxia | 416 |
| Whooper swan | NBC47 | 2015.12.8 | Sanmenxia | 4618 |
| Whooper swan | 2020 | 2015.12.8 | Sanmenxia | 1610 |
| Whooper swan | 1021 | 2015.12.8 | Sanmenxia | 1918 |
| Whooper swan | 2013 | 2015.12.8 | Sanmenxia | 1913 |
| Whooper swan | 2038 | 2015.12.8 | Sanmenxia | 569 |
| Whooper swan | 2039 | 2015.12.8 | Sanmenxia | 1604 |
