# Supplementary material for: New evidence for the east–west spread of the highly pathogenic avian influenza H5N1 virus between Central Asian and east Asian-Australasian flyways in China
Source: Emerg Microbes Infect. 2019 Jun 5;8(1):823–6. doi: 10.1080/22221751.2019.1623719 (PMC6567254; doi:10.1080/22221751.2019.1623719)
Supplement: Supplemental Material [file TEMI_A_1623719_SM1171.zip › Supplementary Material/temi-2018-0039-20190522173938/doc/Supplementary Tables final.zip!/Supplementary Table S2.docx]

Table S2 Accession number for the H5N1 sequences reported in this paper

|  | A/bar-headed goose/Qinghai/133/2015 | A/bar-headed goose/Qinghai/47/2015 | A/bar-headed goose/Qinghai/70/2015 | A/bar-headed goose/Qinghai/F/2015 |
| --- | --- | --- | --- | --- |
| HA | MK641362 | MK641370 | MK641378 | MK641386 |
| NA | MK641364 | MK641372 | MK641380 | MK641388 |
| M | MK641363 | MK641371 | MK641379 | MK641387 |
| NP | MK641365 | MK641373 | MK641381 | MK641389 |
| NS | MK641366 | MK641374 | MK641382 | MK641390 |
| PA | MK641367 | MK641375 | MK641383 | MK641391 |
| PB1 | MK641368 | MK641376 | MK641384 | MK641392 |
| PB2 | MK641369 | MK641377 | MK641385 | MK641393 |
