# Supplementary material for: New evidence for the east–west spread of the highly pathogenic avian influenza H5N1 virus between Central Asian and east Asian-Australasian flyways in China
Source: Emerg Microbes Infect. 2019 Jun 5;8(1):823–6. doi: 10.1080/22221751.2019.1623719 (PMC6567254; doi:10.1080/22221751.2019.1623719)
Supplement: Supplemental Material [file TEMI_A_1623719_SM1171.zip › Supplementary Material/temi-2018-0039-20190522173938/doc/Supplementary Tables final.zip!/Supplementary Table S3.docx]

**Supplementary Table S3 Nucleotide sequence identity levels among the AIVs isolated from Qinghai Lake in 2015 and other AIVs**

| Gene | A/bar-headed goose/Qinghai/F/2015(H5N1) | | | A/bar-headed goose/Qinghai/47/2015(H5N1) | | | A/bar-headed goose/Qinghai/70/2015(H5N1) | | | A/bar-headed goose/Qinghai/133/2015(H5N1) | | |
| --- | --- | --- | --- | --- | --- | --- | --- | --- | --- | --- | --- | --- |
|  | Reference strain | SN | Homology | Reference strain | SN | Homology | Reference strain | SN | Homology | Reference strain | SN | Homology |
| PB2 | SMX3 | EPI559761 | 99.91% | SMX1 | EPI559724 | 99.82% | SMX1 | EPI559724 | 99.69% | SMX3 | EPI559761 | 99.86% |
| PB1 | SMX4 | EPI559800 | 100% | SMX4 | EPI559800 | 100% | SMX4 | EPI559800 | 99.95% | SMX4 | EPI559800 | 100% |
| PA | SMX1 | EPI560206 | 99.76% | SMX1 | EPI560206 | 99.72% | SMX1 | EPI560206 | 99.76% | SMX1 | EPI560206 | 99.72% |
| HA | SMX9 | EPI559910 | 99.8% | SMX9 | EPI559910 | 99.8% | SMX9 | EPI559910 | 99.8% | SMX9 | EPI559910 | 99.8% |
| NP | SMX4 | EPI559806 | 100% | SMX4 | EPI559806 | 100% | SMX4 | EPI559806 | 100% | SMX4 | EPI559806 | 100% |
| NA | SMX1 | EPI560203 | 100% | SMX1 | EPI560203 | 100% | SMX1 | EPI560203 | 100% | SMX1 | EPI560203 | 100% |
| M | SMX9 | EPI559928 | 99.9% | SMX9 | EPI559928 | 99.9% | SMX9 | EPI559928 | 99.9% | SMX9 | EPI559928 | 99.9% |
| NS | SMX9 | EPI559931 | 100% | SMX9 | EPI559931 | 99.8% | SMX9 | EPI559931 | 100% | SMX9 | EPI559931 | 100% |

SMX1:A/whooper swan/Henan/SMX1/2015(H5N1)(SMX1); SMX3: A/whooper swan/Henan/SMX3/2015(H5N1)(SMX3); SMX4: A/whooper swan/Henan/SMX4/2015(H5N1)(SMX4); SMX9: A/whooper swan/Henan/SMX9/2015(H5N1)(SMX9).
