# Supplementary material for: New evidence for the east–west spread of the highly pathogenic avian influenza H5N1 virus between Central Asian and east Asian-Australasian flyways in China
Source: Emerg Microbes Infect. 2019 Jun 5;8(1):823–6. doi: 10.1080/22221751.2019.1623719 (PMC6567254; doi:10.1080/22221751.2019.1623719)
Supplement: Supplemental Material [file TEMI_A_1623719_SM1171.zip › Supplementary Material/temi-2018-0039-20190522173938/doc/Supplementary Tables final.zip!/Supplementary Table S4.docx]

**Table S4 HA gene sequences of H5N1 virus used in this study**

| Strain | Collection date |
| --- | --- |
| A/chicken/China/JX/2011 | 2011-12-18 |
| A/chicken/AnNing/1/2014 | 2014-01-01 |
| A/environment/Guizhou/2/2009 | 2009-01-16 |
| A/chicken/Tibet/6/2008 | 2008 |
| A/Anhui/1/2005 | 2005-11-01 |
| A/Jiangsu/1/2007 | 2007 |
| A/duck/Hunan/15/2004 | 2004-01-28 |
| A/goose/Guiyang/3422/2005 | 2005 |
| A/barn_swallow/Hong_Kong/1161/2010 | 2010-03 |
| A/black-crowned_night_heron/Hong_Kong/659/2008 | 2008 |
| A/chicken/Guangxi/S2039/2009 | 2009-05-06 |
| A/chicken/Hong_Kong/782/2009 | 2009 |
| A/chicken/Huabei/0513/2007 | 2007-05-13 |
| A/chicken/Hunan/3/2007 | 2007-10 |
| A/chicken/Hunan/8/2008 | 2008-11 |
| A/chicken/Jiangsu/k0402/2010 | 2010-02-10 |
| A/chicken/Korea/Gimje/2008 | 2008-04-01 |
| A/common_buzzard/Hong_Kong/9213/2007 | 2007 |
| A/common_magpie/Hong_Kong/5052/2007 | 2007 |
| A/crested_myna/Hong_Kong/1178/2009 | 2009 |
| A/Daurian_starling/Hong_Kong/1532/2007 | 2007 |
| A/duck/China/G3/2012 | 2012-01-16 |
| A/duck/Hubei/xn/2007 | 2007-11-14 |
| A/duck/Hunan/3/2007 | 2007-10 |
| A/duck/Hunan/8/2008 | 2008-11 |
| A/duck/Hunan/S4020/2008 | 2008-11-26 |
| A/duck/Hunan/S4030/2011 | 2011-11-09 |
| A/duck/Hunan/S4220/2011 | 2011-11-13 |
| A/duck/Hunan/S4234/2011 | 2011-11-13 |
| A/duck/Nanchang/9789/2013 | 2013-10-01 |
| A/duck/Zhejiang/213/2011 | 2011-02 |
| A/duck/Zhejiang/224/2011 | 2011-02 |
| A/duck/Zhejiang/2244/2011 | 2011-02 |
| A/duck/Zhejiang/2245/2011 | 2011-02 |
| A/duck/Zhejiang/2248/2011 | 2011-02 |
| A/feral_pigeon/Hong_Kong/3409/2009 | 2009 |
| A/goose/Jiangsu/k0403/2010 | 2010-02-10 |
| A/great_egret/Hong_Kong/807/2008 | 2008 |
| A/grey_heron/Hong_Kong/1046/2008 | 2008 |
| A/grey_heron/Hong_Kong/3088/2007 | 2007 |
| A/grey_heron/Hong_Kong/779/2009 | 2009 |
| A/house_crow/Hong_Kong/5288/2007 | 2007 |
| A/house_crow/Hong_Kong/7677/2008 | 2008 |
| A/Hubei/1/2010 | 2010-05-21 |
| A/large_billed_crow/Hong_Kong/885/2009 | 2009 |
| A/little_egret/Hong_Kong/8550/2007 | 2007 |
| A/little_egret/Hong_Kong/8863/2007 | 2007 |
| A/magpie_robin/Hong_Kong/1097/2008 | 2008 |
| A/magpie_robin/Hong_Kong/1897/2008 | 2008 |
| A/oriental_magpie_robin/Hong_Kong/9298/2009 | 2009 |
| A/peregrine_falcon/Hong_Kong/1143/2007 | 2007 |
| A/quail/Nanchang/6631/2013 | 2013-10-01 |
| A/red-billed_starling/Hong_Kong/4925/2007 | 2007 |
| A/swan/Shanghai/10/2009 | 2009-01 |
| A/swine/Guangxi/592/2011 | 2011-02-27 |
| A/tiger/Yunnan/tig1412/2014 | 2014-12-15 |
| A/wild_bird/Hong_Kong/07035-1/2011 | 2011-12-13 |
| A/wild_duck/Fujian/1/2011 | 2011-03-10 |
| A/chicken/Guangdong/178/04 | 2004 |
| A/chicken/Guangxi/2448/2004 | 2004 |
| A/chicken/Guangxi/2461/2004 | 2004 |
| A/chicken/Guangxi/3154/2005 | 2005 |
| A/chicken/Guangxi/3791/2005 | 2005 |
| A/chicken/Guangxi/604/2005 | 2005 |
| A/chicken/Guiyang/2147/2005 | 2005 |
| A/Chicken/Shantou/810/05 | 2005 |
| A/chicken/Shantou/810/2005 | 2005 |
| A/chicken/Yunnan/chuxiong01/2005 | 2005 |
| A/Chinese_pond_heron/Hong_Kong/18/2005 | 2005 |
| A/duck/China/E319-2/03 | 2003 |
| A/duck/Guangdong/23/2004 | 2004 |
| A/duck/Guangxi/2926/2005 | 2005 |
| A/duck/Guangxi/3085/2005 | 2005 |
| A/duck/Guangxi/3364/2005 | 2005 |
| A/duck/Guangxi/351/2004 | 2004 |
| A/duck/Guangxi/3548/2005 | 2005 |
| A/duck/Guangxi/3741/2005 | 2005 |
| A/duck/Guangxi/3819/2005 | 2005 |
| A/duck/Guangxi/4016/2005 | 2005 |
| A/duck/Guangxi/4184/2005 | 2005 |
| A/duck/Guangxi/4196/2005 | 2005 |
| A/duck/Guangxi/4665/2005 | 2005 |
| A/duck/Guangxi/793/2005 | 2005 |
| A/duck/Guangxi/89/2006 | 2006 |
| A/duck/Guangxi/951/2005 | 2005 |
| A/duck/Hunan/1265/2005 | 2005 |
| A/duck/Hunan/1608/2005 | 2005 |
| A/duck/Yunnan/1126/2006 | 2006 |
| A/duck/Yunnan/4400/2005 | 2005 |
| A/duck/Yunnan/4589/2005 | 2005 |
| A/duck/Yunnan/5251/2005 | 2005 |
| A/duck/Yunnan/5820/2005 | 2005 |
| A/duck/Yunnan/5877/2005 | 2005 |
| A/duck/Yunnan/6332/2005 | 2005 |
| A/duck/Yunnan/6607/2005 | 2005 |
| A/goose/Guangxi/3017/2005 | 2005 |
| A/goose/Guangxi/3316/2005 | 2005 |
| A/goose/Guangxi/345/2005 | 2005 |
| A/goose/Guangxi/3714/2005 | 2005 |
| A/goose/Yunnan/3720/2005 | 2005 |
| A/goose/Yunnan/4129/2005 | 2005 |
| A/goose/Yunnan/4494/2005 | 2005 |
| A/goose/Yunnan/4804/2005 | 2005 |
| A/goose/Yunnan/6027/2005 | 2005 |
| A/goose/Yunnan/6368/2005 | 2005 |
| A/grey_heron/Hong_Kong/728/2004 | 2004 |
| A/grey_heron/Hong_Kong/837/2004 | 2004 |
| A/peregrine_falcon/Hong_Kong/5211/2006 | 2006 |
| A/Ph/ST/44/2004 | 2004 |
| A/pheasant/Shantou/2239/2006 | 2006 |
| A/pika/Qinghai/GRL/2007 | 2007-12-04 |
| A/pika/Qinghai/QW/2007 | 2007-12-05 |
| A/pika/Qinghai/SHK/2007 | 2007-12-02 |
| A/quail/Guangxi/575/2005 | 2005 |
| A/Quail/Shantou/911/05 | 2005 |
| A/silky_chicken/Shantou/475/2004 | 2004 |
| A/duck/Hubei/3/2005 | 2005 |
| A/duck/Hunan/127/2005 | 2005 |
| A/Bangladesh/207095/2008 | 2008-02 |
| A/chicken/Israel/65/2010 | 2010-01-25 |
| A/chicken/Egypt/1036/2010 | 2010-02 |
| A/Indonesia/8228/2008 | 2008 |
| A/Indonesia/625/06 | 2006 |
| A/chicken/Indonesia/11/2003 | 2003-12-20 |
| A/chicken/Henan/210/2004 | 2004 |
| A/duck/Guangxi/50/2001 | 2001 |
| A/chicken/HK/61.9/02 | 2002 |
| A/chicken/HK/YU22/2002 | 2002 |
| A/chicken/Shanxi/10/2006 | 2006 |
| A/chicken/Shanxi/2/2006 | 2006 |
| A/goose/Yunnan/3315/2005 | 2005 |
| A/blackbird/Hunan/1/2004 | 2004 |
| A/duck/Hubei/wg/2002 | 2002 |
| A/chicken/Jilin/ho/2003 | 2003 |
| A/duck/Guangxi/1378/2004 | 2004 |
| A/duck/Guangdong/22/2002 | 2002 |
| A/goose/Guiyang/337/2006 | 2006 |
| A/chicken/Hebei/718/2001 | 2001 |
| A/duck/Guangxi/xa/2001 | 2001 |
| A/chicken/Guangdong/174/04 | 2004 |
| A/chicken/Korea/ES/03 | 2003 |
| A/chicken/Guangxi/12/2004 | 2004 |
| A/duck/Yunnan/4645/2003 | 2003 |
| A/Cambodia/U0417030/2010 | 2010-04-18 |
| A/Vietnam/UT3030/2003 | 2003-12-29 |
| A/Chicken/Hong_Kong/y388/97 | 1997 |
| A/goose/Guangdong/1/96 | 1996 |
| A/tiger/Jiangsu/01/2013 | 2013-01-20 |
| A/Japanese_white-eye/Taoyuan/Q454/2012 | 2012-07-12 |
| A/duck/Wenzhou/YJYF33/2015 | 2015-01 |
| A/duck/Wenzhou/HAYXLG10/2015 | 2015-01 |
| A/chicken/Wuhan/HAQL07/2014 | 2014-12 |
| A/chicken/Wuhan/HAQL16/2014 | 2014-12 |
| A/goose/Zhejiang/112071/2014 | 2014-11-20 |
| A/pigeon/Zhejiang/112089/2014 | 2014-11-20 |
| A/pigeon/Zhejiang/112090/2014 | 2014-11-20 |
| A/duck/Zhejiang/1220092/2014 | 2014-12-20 |
| A/duck/Hunan/S4150/2011 | 2011-11-11 |
| A/pigeon/Zhejiang/727097/2014 | 2014-07-27 |
| A/Great_black-headed_Gull/Qinghai/YO1/2015 | 2015-07-14 |
| A/bar-headed_goose/Qinghai/47/2015 | 2015-07-06 |
| A/bar-headed_goose/Qinghai/70/2015 | 2015-07-06 |
| A/bar-headed_goose/Qinghai/133/2015 | 2015-07-06 |
| A/great_black-headed_gull/Qinghai/2/2009 | 2009-05 |
| A/great_black-headed_gull/Qinghai/6/2009 | 2009-05-28 |
| A/great_cormorant/Qinghai/1/2009 | 2009-05 |
| A/brown-headed_gull/Qinghai/1/2009 | 2009-05 |
| A/brown-headed_gull/Qinghai/9/2009 | 2009-06 |
| A/brown-headed_gull/Qinghai/19/2009 | 2009-06 |
| A/great_black-headed_gull/1/2009 | 2009-05 |
| A/great_black-headed_gull/Qinghai/3/2009 | 2009-05-18 |
| A/great_black-headed_gull/Qinghai/4/2009 | 2009-05-18 |
| A/great_black-headed_gull/Qinghai/5/2009 | 2009-05 |
| A/brown-headed_gull/Qinghai/2/2009 | 2009-05 |
| A/great_crested-grebe/Qinghai/1/2009 | 2009-05-08 |
| A/bar-headed_goose/Qinghai/1/2010 | 2010-05 |
| A/great_black-headed_gull/Qinghai/8/2009 | 2009-06 |
| A/great_crested_grebe/Qinghai/1/2010 | 2010-05 |
| A/wild_waterfowl/Shaanxi/F91/2015 | 2015-03-18 |
| A/Black-necked_Grebe/Inner_Mongolia/Brain1/2015 | 2015-05-12 |
| A/whooper_swan/Henan/SMX9/2015 | 2015-01-05 |
| A/whooper_swan/Henan/SMX3/2015 | 2015-01-04 |
| A/enviroment/Henan/SMX1/2015 | 2015-01-05 |
| A/whooper_swan/Henan/SMX1/2015 | 2015-01-04 |
| A/whooper_swan/Henan/SMX4/2015 | 2015-01-05 |
| A/pochard/Henan/SMX1/2015 | 2015-01-05 |
| A/bar-headed_goose/Qinghai/F/2015 | 2015-07-06 |
| A/hill_myna/Heilongjiang/0704/2012 | 2012-07-04 |
| A/Hong_Kong/6841/2010 | 2010-11-16 |
| A/chicken/Hubei/RA18/2010 | 2010-10-17 |
| A/chicken/Jiangsu/2477/2014 | 2014-06-17 |
| A/chicken/Jiangsu/927/2013 | 2013-11-18 |
| A/wild_duck/Jilin/HF/2011 | 2011-04-25 |
| A/wild_duck/Jilin/ZF/2011 | 2011-04-26 |
| A/wild_duck/Shandong/2/2011 | 2011-04-13 |
| A/whooper_swan/Shanxi/17L/2015 | 2015-01-06 |
| A/peacock/Yunnan/1/2015 | 2015-06-02 |
| A/peacock/Yunnan/3/2015 | 2015-06-02 |
| A/tiger/Yunnan/tig1508/2015 | 2015-08-12 |
